# Exploring synergies between B- and T-cell vaccine approaches to optimize immune responses against HIV—workshop report

**DOI:** 10.1038/s41541-024-00818-y

**Published:** 2024-02-21

**Authors:** Milton Maciel, Rama R. Amara, Katharine J. Bar, Shane Crotty, Steven G. Deeks, Christopher Duplessis, Gaurav Gaiha, M. Juliana McElrath, Andrew McMichael, Amy Palin, Rachel Rutishauser, Stuart Shapiro, Stephen T. Smiley, M. Patricia D’Souza

**Affiliations:** 1 Division of AIDS, National Institute of Allergy and Infectious Diseases, National Institutes of Health, Rockville, MD USA; 2 Yerkes National Primate Research Center, Emory University, Atlanta, GA, USA; 3 Department of Medicine, University of Pennsylvania, Philadelphia, PA USA; 4 Center for Infectious Disease and Vaccine Research, La Jolla Institute for Immunology (LJI), La Jolla, CA 92037 USA; 5 Department of Medicine, Division of Infectious Diseases and Global Public Health, University of California, San Diego (UCSD), La Jolla, CA USA; 6 Division of HIV, Infectious Diseases, and Global Medicine, San Francisco, CA, USA; 7 Department of Medicine, University of California at San Francisco, San Francisco, CA USA; 8 Ragon Institute of Mass General, MIT and Harvard, Cambridge, MA USA; 9 Vaccine and Infectious Disease Division, Fred Hutchinson Cancer Research Center, Seattle, WA USA; 10 Nuffield Department of Clinical Medicine, University of Oxford, Oxford, UK; 11 Department of Medicine, University of California, San Francisco, CA USA

**Keywords:** Vaccines, RNA vaccines

## Abstract

The US National Institute of Allergy and Infectious Diseases (NIAID), part of the National Institute of Health (NIH), convened a virtual workshop on August 8-9^th^, 2023 to explore potential synergies between HIV vaccine approaches that are designed to induce cellular or humoral immune responses. The goal of this workshop was to review data on leading vaccine candidates and to discuss the best strategies for combining these approaches to optimize immunity against HIV. Here, we summarize the findings reviewed at the workshop and discuss the knowledge gaps and priorities for future studies that will help accelerate the development of a preventive HIV vaccine.

## Why are combination vaccine approaches necessary?

There are many challenges to the design and development of a broadly effective HIV vaccine. Chief among them are HIV’s genetic variability and the virus’s extensive immune evasion tactics.

Over the past four decades researchers have pursued multiple vaccine strategies to address these challenges, including the most recent large-scale efficacy trials of epitope mosaic vaccine candidates designed specifically to address HIV’s extraordinary global diversity^1^. None of the vaccine regimens tested to date have induced broad and durable protection^2,3^. Out of nine HIV-1 vaccine efficacy trials completed so far, only one demonstrated correlates of protection^4^. The only trial that showed a small degree of estimated efficacy in reducing HIV-1 transmission (31.2%) was the RV144 HIV-1 (NCT00223080) trial of the CRFAE_01 canarypox/gp120 vaccine in Thailand. However, of three phase IIb/III clinical trials designed to improve on the RV144 trial — HIV-1 Vaccine Trials Network (HVTN) 702 (NCT02968849), HVTN 705 (NCT03060629) and HVTN 706 (NCT03964415), none showed significant efficacy, suggesting that the RV144 trial may not have been a harbinger of vaccine success.

Meanwhile, results from the recently completed Antibody Mediated Prevention (AMP) trials showed that passive administration of broadly neutralizing antibodies (bNAbs) with sufficient specificity can protect against HIV infection by neutralization-sensitive strains when serum antibody levels were present at high enough concentration^5^. The AMP results provide an important proof of concept for the role of bNAbs in protecting against HIV. Thus, for HIV-1, the induction of antibodies that broadly protect against heterologous HIV-1 strains is a prime goal of HIV-1 vaccine development. The results suggest a high bar for protective immunity through bNAbs alone, according to Shane Crotty of the La Jolla Institute for Immunology, who provided opening remarks at the workshop.

Preclinical studies corroborate this. It has been shown that vaccine-elicited antibodies can protect 90% of non-human primates (NHPs) against rectal challenge with an simian-human immunodeficiency virus (SHIV), but only at high titers of circulating neutralizing antibodies (greater than 1:500)^6^.

Given this high bar for protection based only on neutralizing antibodies, researchers are increasingly interested in combining B and T cell vaccine strategies to establish protection at, more attainable, lower concentrations of bNAbs. It seems reasonable to hypothesize that bNAbs can confer sterilizing protection, while T cell-based immunity will control and/or eliminate cells infected by virions escaping neutralization.

This hypothesis was borne out in recent preclinical studies. Combining an HIV SOSIP protein vaccine candidate administered with a TLR7/TLR8-agonist adjuvant (i.e. 3M-052) that induces autologous neutralizing antibodies with potent T cell-inducing viral vectors provided better protection against homologous SHIV challenge in rhesus macaques than either candidate on its own. In fact, the combination antibody and T cell vaccine was protective even with lower, sub-optimal titers of neutralizing antibodies^7^. Similarly, vaccine-mediated induction of a potent T cell response lowered the antibody threshold needed to prevent SARS-CoV-2 infection in non-human primates^8^. The precise mechanisms for these effects remain unknown.

This observation, the results from the AMP trials, and the ongoing clinical evaluations of promising B and T cell-based vaccine candidates investigated with novel clinical designs as described by Hahn et al.^9^ and Table 1, allied to investigations of the effect of bNAbs and/or T cell-based vaccines in people with HIV (PWH) (see below) are inspiring researchers to explore potential synergies between these strategies. The virtual workshop convened by the US National Institute of Allergy and Infectious Diseases (NIAID) on August 8-9^th^ 2023 was a key step in facilitating these efforts.

**Table 1:** HVTN current and future phase 1 and discovery medicine clinical trials for investigation of HIV vaccine candidates for generation of broad neutralizing antibodies or T cell responses.

Trial (NCT number)	Immunogen (platform)	Adjuvant(s)	Target epitope(s)
**HVTN 144 (NCT06033209)**	N332-GT5 gp140 (Protein)	SMNP	V3 glycan
**HVTN 300 (NCT04915768)**	CH505 TF chTrimer (protein)	3M-052-AF +/- Alum, Empty LNP	CD4bs, V2 apex, V3 glycan
**HVTN 301 (NCT05471076)**	426 c.Mod.Core-C4b (protein)	3M-052-AF + Alum	CD4bs
**HVTN 302(NCT05217641)**	・BG505 MD39.3 trimer soluble (mRNA) ・BG505 MD39.3 gp151 trimer membrane-bound (mRNA) ・BG505 MD39.3 gp151 CD4KO trimer (mRNA)	–	V3 complex N-linked glycans, N332-dependent
**HVTN 304(NCT05828095)**	・HIV Env Trimer/IL-12 (INO-6160) (DNA) ・4571 Trimer (BG505) (protein)	3M-052-AF + Alum	Binding/neutralizing AbsCD4/CD8 responses
**HVTN 305(NCT05781542)**	・sD-NP-GT8/IL-12 (DNA) ・Trimer 4571 (BG505) (protein)	3M-052-AF + Alum	CD4bs
**HVTN 307(NCT05903339)**	V3G CH848 Pr-NP1 (ferritin nanoparticle as protein) V3G CH848 mRNA-Tr2 (mRNA encoding ferritin nanoparticle)	Empty LNP 3M-052-AF	V3 glycan
**HVT 142 (NCT05854381)**	VIR 1388 expressing HIV Mfuse1(human CMV vector)	–	Gag, Pol, Nef T cell epitopes
HVTN 309	・CD4BS CH505M5 Pr-1 (RNA) ・CH505 chTrimer (protein)	Empty LNP 3M-052-AF	CD4bs, V2 apex, V3 glycan
HVTN 310	HIV-1 Env, HIV-1 Gag and HIV-1 Gag/Pro (mRNA for in vivo VLP generation)	–	CD4bs, V2 apex
HVTN 312	Prime: CH505 M5 N197D gp160 (mRNA) Boost: CH505 TF gp160 (mRNA)	–	Early CH235 lineage intermediates ・CH505 TF boost can increase improbable mutations

*LNP* lipid nanoparticle, *TF* transmitter founder, *sD-NP-GT8* synthetic DNA-encoded nanoprotein GT8, *SMNP* saponin/MPLA nanoparticles, *3M-052-AF* 3M-052 aqueous formulation, *FP8v1-rTTHC* 8 amino acids at N term of HIV fusion protein conjugated to tetanus toxoid heavy chain fragment C, *VLP* virus like-particle.

Bold rows show clinical trials in preparation.

Experts at the workshop reviewed the leading approaches to inducing both cellular and humoral immunity and identified major gaps in knowledge and opportunities for collaborative research to evaluate and optimize combined vaccine regimens and thereby accelerate the development of a preventive HIV vaccine.

## The landscape of B cell-targeted vaccines

Broadly neutralizing antibodies develop only rarely in a minority of natural HIV infection and have proven difficult to induce through vaccination; however, researchers are heavily focused on this effort. One of the most promising strategies for inducing bNAbs through vaccination involves mimicking the elaborate process of antibody evolution that naturally occurs in the small subset of HIV-infected individuals following multiple rounds of somatic hypermutation and selection in response to an ever-evolving virus^4^.

To achieve similar responses through vaccination, researchers are exploring multiple strategies, including germline-targeting and B-cell lineage approaches^4^. Germline targeting employs structure-based design to reverse engineer HIV immunogens that can first bind naïve B cells, followed by additional immunogens that can trigger further affinity maturation that will eventually give rise to potent bNAbs. B cell lineage approaches computationally reconstruct the maturation history of a bNAb from an HIV-infected individual and employs this as a guide for sequential immunizations^10^. Both strategies require a series of immunizations with increasingly native-like HIV Envelope immunogens to shepherd the immune system to induce specific types of highly mutated bNAbs^4^.

Barton Haynes of the Duke University and Dennis Burton of the Scripps Research each lead a Consortium for HIV/AIDS Vaccine Development (CHAVD) program funded by the NIH. At the workshop, they both presented their plans for clinical trials to test and optimize vaccine regimens to induce bNAbs (Ref. ^8^, Table 1).

Burton discussed an engineered nanoparticle vaccine immunogen referred to as eOD-GT8 60 mer, which was developed by scientists at Scripps Research, Fred Hutchinson Cancer Center (through the HIV Vaccine Trial Network, HVTN), NIAID, and IAVI and tested in Phase 1 clinical trials. The vast majority of vaccinees (97%) showed VRC-01-class bNAb precursors with a median frequency of 0.1% IgG^+^ B cells in blood, indicating that reverse immunogen engineering can stimulate and expand rare germline B cells^11,12^.

Researchers are also testing the eOD-GT8 immunogen expressed from mRNA encapsulated in lipid nanoparticles (IAVI G002, NCT05001373), and, according to Burton, this approach appears even more promising than the equivalent nanoparticle protein previously tested^12^. The goal now is to continue this work and to test eOD-GT8 and other germline-targeting immunogens singly and in combination with more native-like stabilized HIV envelope trimers. i.e. SOSIPs^13^. Currently, HVTN 302 phase 1 clinical trial (NCT05217641) is evaluating safety and immunogenicity of the stabilized HIV Env trimers delivered as mRNA (Table 1). According to Burton, a reasonable goal for the field is to advance one full sequential immunization protocol that proves successful in a stringent preclinical model into human clinical trials within the next three years.

One way to simplify the development and testing of these immunogens in iterative early-stage clinical trials is to use mRNA platforms. The flexibility of mRNA platforms, which proved highly successful during the COVID-19 pandemic, offers numerous advantages, chiefly, the time between design and manufacture of clinical-grade material. In addition to its speed and versatility, mRNA vaccines are shown to induce high levels of T follicular helper (Tfh) cells, as seen in experimental models^14^, as well as by quantification of antigen-specific responses in draining axillary lymph node samples^15^, which, at high and sustained levels, are essential to eliciting optimal bNAb responses, according to Haynes^16^. His group is planning several clinical trials to also evaluate the adjuvant effect of empty lipid nanoparticles on HIV protein immunogens (Table 1).

## Striking the right balance

Stimulation of Tfh cells is just one of many ways B and T cells vaccine platforms may work in tandem to enhance immunity. As outlined by Crotty, there are multiple mechanisms for synergy between B and T cells, including the induction of local innate immunity, increased local antibody concentration following CD8^+^ T_RM_-induced tissue permeability^17^, and the help CD4^+^ T cells provide to both B and CD8^+^ T cells.

However, HIV vaccines that lead to strong activation of CD4^+^ T cell responses may be a double-edged sword, as some participants noted. If CD8^+^ T cells are not present at sufficient levels, activated CD4^+^ T cells induced by a vaccine may augment HIV infection risk, as was seen in the STEP trial^18^, and in NHP studies, as pointed out by Paolo Lusso of NIAID^19^. Optimization of assays to interrogate HIV susceptibility induced by viral vector, as seen by in vitro studies where the preexisting response to adenovirus serotype 5 led to preferential expansion of HIV-susceptible activated CD4^+^ T cells^20^, are necessary for future early-phase clinical trials.

Decades of work studying HIV controllers—PWH who maintain undetectable to low viral loads without antiretroviral therapy, has provided valuable clues about the features of CD8^+^ T cell responses that are most beneficial for virus control. Bruce Walker of the Ragon Institute said that CD8^+^ T cell proliferation and specificity are the factors most consistently associated with control of HIV. Walker and colleagues have shown that the breadth of CD8^+^ T cell responses to HIV Gag protein are associated with lower viral loads^21^, a finding that was also seen in the HVTN 505 trials, reviewed by Steve DeRosa of the Fred Hutchinson Cancer Research Center and the HVTN. According to Mark Connors of NIAID, the most important function of CD8^+^ T cells is their antiviral activity. He emphasized that assays to measure T cell functions need to be tied more closely to antiviral response^22^.

## Evidence for synergy

One main focus of the workshop was the key role that CD8^+^ T cells may play in recruiting B cells into tissues. In a preclinical study led by Bali Pulendran of Stanford University, researchers showed that a BG505 SOSIP.664 trimer immunogen that induced tier-2 neutralizing antibody responses could protect against low-dose mucosal SHIV challenge in rhesus macaques at a serum antibody titer of 1:319^7^. However, when this B cell immunogen was combined with a vaccine regimen that has been shown to induce high levels of CD8^+^ T cells in vaginal tissues^23^, the titer of neutralizing antibodies required for protection was reduced. Combined B and T cell vaccine regimens also enhanced the durability of protection.

**Fig. 1:**
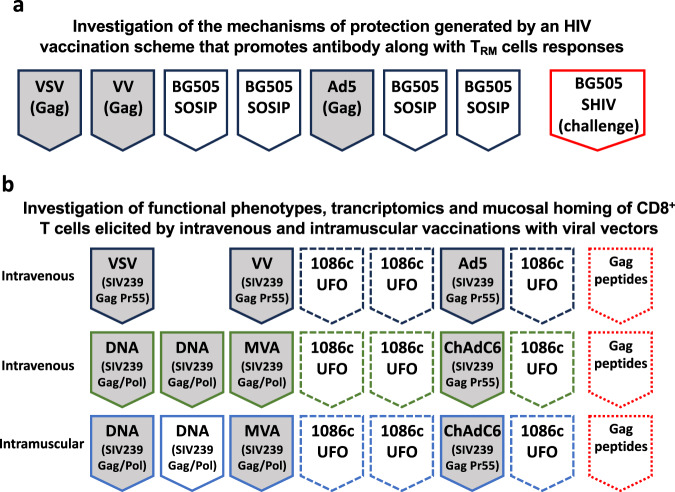
Non-human primate studies ongoing at Emory to investigate the mechanisms of collaboration between T and B cell HIV vaccines. Schemes were simplified to indicate the products and sequence of immunization; control groups, intervals and doses are not indicated. **a** Immunizations performed via intramuscular injections; heterologous viral vectors: *VSV* Vesicular Stomatitis virus, *VV* vaccinia virus, *Ad5* Adenovirus type 5. BG505 SOSIP trimer - stabilized envelope protein (administered with 3M-052-AF+Alum). Red box – simian-human immunodeficiency virus challenges administered intravaginally. **b** Immunization routes are indicated on the left of each group; heterologous viral vectors: *VSV* Vesicular Stomatitis virus, *VV* vaccinia virus, *Ad5* Adenovirus type 5, *MVA* modified vaccinia Ankara, *ChAdC6* chimpanzee Adenovirus type C6. 1086c UFO – soluble uncleaved pre-fusion optimized gp140 trimer protein (administered subcutaneously with 3M-052-PLGA nanoparticles). Dotted red boxes - Gag peptide – immunodominant Gag peptide recognized by MAMU-A-1 are delivered vaginally non-traumatically 24 hs prior to tissue collection. Dashed boxes indicate intramuscular administration of the 1086c UFO protein in all groups.

Follow-up studies to confirm synergies between B and T cell vaccines in NHPs are already underway, as presented by Rama Amara of Emory University (Fig. 1). In the aforementioned NHP study by Pulendran’s group, T cell response was elicited with a sequential regimen of three different viral vectors delivered intravenously. Experts agreed that meticulous comparative preclinical studies evaluating multiple viral vector-based T cell vaccine candidates in combination with sub-optimal levels of passively administered bNAbs are necessary to identify more practical regimens for clinical trials.

According to Gaurav Gaiha of the Ragon Institute, adenoviral vectors and RNA replicons induce important T cell responses, and several attendees asserted that live-viral vector-based vaccine candidates offer several advantages, such as their ability to persist and create durable T cell immunity at multiple sites.

## The advantages of the cytomegalovirus vector

One of the most promising live viral vectors in development is the attenuated cytomegalovirus (CMV) vaccine. Several studies have shown that the rhesus CMV-based SIV vaccine developed at Oregon Health & Science University (OHSU) can reproducibly prevent viral replication in more than half of rhesus macaques who were vaccinated and then exposed to repeated mucosal challenges^24^.

Klaus Fruh of OHSU postulated that the CMV vectors work because they prevent viral spread during acute infection and prevent the establishment of the latent reservoir of SIV-infected cells, similar to how antiretroviral therapy administered early after infection can prevent the latent infection^25^.

He also showed that the serendipitous deletion of select CMV genes elicits CD8^+^ T cells restricted by the MHC class Ib molecule HLA-E^26,27^, and that this unconventional response is required for the observed efficacy of the vaccine. Jonah Sacha, also of OHSU, presented data showing that a cynomolgus macaque CMV vector with the same gene deletions also induced HLA-E-restricted T cell responses, which further indicates that this property is intrinsic to the vector. Additional studies have implicated baseline levels of interleukin (IL)-15 signaling—a known regulator of CD8^+^T cell function—as inversely correlated with the efficacy of this vaccine construct^28^.

There was great interest in further exploring the role of HLA-E -restricted CD8^+^ T cells in protection against HIV. It is still unknown whether a human CMV vector will induce these unconventional responses, but according to Andrew McMichael of the Oxford University it should be possible for vaccines to stimulate HIV-specific, HLA-E-restricted T cells.

One question debated at the workshop was how many epitopes in an HIV vaccine immunogen would need to bind HLA-E to adequately address the diversity of the virus. Paul Goepfert of the University of Alabama at Birmingham argued that the population diversity of HIV will likely require an increased breadth of epitopes even with an HLA-E-restricted vaccine. Sacha and others seemed optimistic this was possible as several HIV epitopes bind HLA-E and viewed this as an important advantage of targeting this type of T-cell response. Assays are available to assess which peptides bind to HLA-E^29^, however, Geraldine Gillespie of Oxford pointed out that the assays need to be qualified before they can be considered for assessment of clinical samples.

VIR Biotechnology, Inc. is now evaluating a human CMV-based HIV vaccine construct in early-stage clinical trials. The first Phase I trial (NCT04725877) showed their CMV-HIV vaccine candidate was safe and generally well tolerated. Another Phase I trial, HVTN 142, is ongoing (see Table 1). According to Ann Arvin of VIR, the CMV vector being tested in the HVTN 142 trial, VIR 1388, has several gene deletions that could potentially enhance HLA-E-restricted CD8^+^ T cell responses.

Researchers are eager to explore the CMV vector in combination with different concentrations of passively administered neutralizing antibodies to test the added protection conferred by T cell responses. In fact, Fruh and colleagues have partnered with Burton to test the rhesus CMV-vectored SIV vaccine candidates in combination with sub-optimal levels of passively administered bNAbs to see whether the combination enhances the efficacy of the CMV vaccine. It is possible that, in addition to directly killing virus-infected cells and enhancing local innate immune responses, CD8^+^ resident memory T cells may locally elevate bNAb concentrations, as previously proposed^17^.

## Research gaps and opportunities

In addition to identifying the optimal T cell vaccine vectors and immunogens, the group noted several other important knowledge gaps that should be addressed to advance the field’s understanding of potential synergies between B and T cell vaccine approaches.

One of these gaps is identifying optimal methods for collecting and measuring T cell responses in tissues. Many speakers noted that efforts to study tissue immunity are in their infancy, highlighting difficulties in normalizing between sample collections. Given this, there was discussion about the best strategies for collecting cells from mucosal tissues and the importance of identifying new methods and functional assays for assessing these responses.

It is difficult to sample mucosal sites without disrupting the tissues and skewing the results in animal studies, according to Lusso. Sampling human mucosal tissues is even more complex, and it is unclear whether it is best to measure responses using mucosal secretions, fine needle aspirates from lymph nodes, or local biopsies. Researchers agreed that identifying proxy markers in the blood for T cell activity in the mucosa would be ideal.

In the absence of such markers, tissue sampling will likely remain necessary. Sarah Andrews of NIAID showed that germinal center B cells collected by fine needle aspirates in the Phase I trial of the eOD-GT8 60mer immunogen differed from those obtained from peripheral blood. In fact, lymph nodes-derived B cells showed more somatic mutations. She also noted that the laboratorial interrogation of these cells is technically laborious and expensive, which makes this type of sampling only feasible in a subset of clinical trial volunteers.

There was also consensus that more integrated analyses across cell types will be required to better understand synergies between different vaccine strategies. Julie McElrath of the Fred Hutchinson Cancer Research Center and HVTN conveyed that the field needs to identify methods for assessing the synergy between B and T cells that are reliable, reproducible, and specific.

Another gap is understanding how the route of administration may affect the immune responses induced by these combined approaches. According to Vineet Joag of the University of Minnesota intravenous vaccination may be optimal for generating tissue-resident memory T cells in NHPs, however this may be vector-dependent. Determining which route of administration provides the best response is another area for further research and optimization. Researchers will also need to know how to bolster the durability of both B and T cell immune responses, which may vary with route of administration, vaccine vector, and adjuvant.

## Opportunities to learn from people with HIV

Finally, researchers discussed how clinical trials enrolling PWH may inform the design of preventive HIV vaccines. Jonathan Li of Harvard Medical School reviewed evidence that plasma antibodies from PWH on antiretroviral therapy (ART) can neutralize HIV derived from their pre-treatment plasma but typically fail to neutralize viruses that rebound upon ART interruption^30^. Nevertheless, autologous antibodies emerge and persist during ART^30–32^, and the presence of such antibodies has been associated with post-treatment control^33^. Boris Juelg of the Ragon Institute showed rebound occurs in some PWH even after infusion with a combination of three potent monoclonal bNAbs. He discussed an ongoing study (NCT04983030) evaluating whether an Ad26/MVA vaccine regimen designed to induce robust T cell responses will complement bNAb infusions, a common theme of this meeting.

Christian Brander of IrsiCaixa AIDS Research Institute discussed trials of vaccine regimens designed to expand HIV-specific T cells in PWH. He showed the magnitude of vaccine-specific T cell response correlates with delays in the time to rebound upon ART interruption, but virus ultimately rebounds in most trial participants^34,35^. An ongoing trial will evaluate whether the addition of a SOSIP immunogen designed to augment bNAb responses can improve the efficacy of a T cell-based vaccine regimen (NCT05208125).

Rachel Rutishauser of the University of California, San Francisco (UCSF) described a small proof of concept study that appeared to induce better control of viral rebound in PWH through the use of a regimen that included T cell-based vaccines, bNAb infusions, and an immunomodulatory TLR9 agonist (NCT04357821). Steven Deeks from UCSF noted multiple clinical trials suggest T cell-based vaccines will be insufficient on their own, but a higher percentage of trial participants appear to maintain lower viral loads after ART interruption when they receive bNAb infusions. Katharine Bar of the University of Pennsylvania suggested the timing of bNAb infusion may critical, with infusions being most advantageous during high antigenic states, such as when ART is first initiated or after viral rebound.

According to Mark Connors of the National Institute of Allergy and Infectious Diseases, replicating vaccine vectors, which produce high antigen loads for a sustained period, might better reflect natural infection and hence might be key to generating robust and durable T and B cell responses.

## Conclusion and next steps

Following the rapid and highly successful development of vaccines against SARS-CoV-2, it is important to note that HIV is one of, if not the most challenging virus vaccinologists have ever faced. While the main target for the current SARS-CoV-2 vaccines - the S protein, accumulate modifications that can slowly affect vaccine efficacy, the envelope protein on the surface of HIV is highly variable, with several sites of glycosylation, and present in low density^36^. However, researchers are encouraged by recent progress in developing both B and T cell-focused strategies for HIV vaccines, as well as by the outcomes of preclinical studies that indicate combining these strategies may offer synergies that can be exploited to reach efficacy^37,38^.

The workshop highlighted critical next steps for the field. Efforts to optimize viral vectors, immunogens, and adjuvants to induce the most effective and persistent T cell responses in tissues, and to test these in combination with passively administered bNAbs in preclinical models are top priority (Table 2). These studies will provide critical information about the best combination strategies to advance into human clinical testing.

**Table 2:** Workshop summary.

Highlights	Next steps
■ Preliminary NHP studies suggest that the combination of T and B cell responses can protect from SHIV challenge; ■ Broad neutralizing Ab precursors were elicited in phase 1 clinical trials (e.g. IAVI 001^a^, HVTN 137^b^, HVTN 301, HVTN 302; Table 1); ■ Several bnAb precursor-eliciting immunogens are being investigated (see Table 1); ■ Platforms to elicit cellular responses, such as CMV-vectored HIV Ags, are in early stage clinical trials (e.g. VIR 1388; Table 1) ■ Antibody and cellular responses can extend the time to rebound after ATI in people with HIV.	■ Improve strategies for concurrent generation of bnAbs and T cell responses; ■ Continue the investigation of T cell vaccine strategies such as CMV-based vaccines and networked epitopes; ■ Develop new and improve existing vaccine vectors; ■ Expand understanding of the immune milieu and molecular signatures in long-term controllers; ■ Identification of protective T cell responses/mechanisms, including tissue-resident T cell responses from cure approaches, therapeutic vaccinations, long-term controllers, and/or post-treatment controllers; ■ Define/establish new reagents and assays to interrogate cellular responses; ■ Identify functional CD8^+^ T cell parameters that reliably correlate with vaccine efficacy

^a^NCT03547245.

^b^NCT04177355.

Importantly, before clinical product development, the specific immune response, including epitope specificities, tissue distribution, and molecular signatures of the response should be well investigated prior to optimization in phase 2 of clinical trials. This should be the gateway into phase 3 trials with higher chance of success.

A preventive HIV vaccine will likely require the complex interaction of all arms of the immune system and therefore testing and optimizing these combined strategies is imperative for the field.
