# Author Correction: Exploring synergies between B- and T-cell vaccine approaches to optimize immune responses against HIV—workshop report

**DOI:** 10.1038/s41541-024-00852-w

**Published:** 2024-03-14

**Authors:** Milton Maciel, Rama R. Amara, Katharine J. Bar, Shane Crotty, Steven G. Deeks, Christopher Duplessis, Gaurav Gaiha, M. Juliana McElrath, Andrew McMichael, Amy Palin, Rachel Rutishauser, Stuart Shapiro, Stephen T. Smiley, M. Patricia D’Souza

**Affiliations:** 1 Division of AIDS, National Institute of Allergy and Infectious Diseases, National Institutes of Health, Rockville, MD USA; 2 Yerkes National Primate Research Center, Emory University, Atlanta, GA USA; 3 Department of Medicine, University of Pennsylvania, Philadelphia, PA USA; 4 Center for Infectious Disease and Vaccine Research, La Jolla Institute for Immunology (LJI), La Jolla, CA 92037 USA; 5 Department of Medicine, Division of Infectious Diseases and Global Public Health, University of California, San Diego (UCSD), La Jolla, CA USA; 6 Division of HIV, Infectious Diseases, and Global Medicine, San Francisco, CA USA; 7 Department of Medicine, University of California at San Francisco, San Francisco, CA USA; 8 Ragon Institute of Mass General, MIT and Harvard, Cambridge, MA USA; 9 Vaccine and Infectious Disease Division, Fred Hutchinson Cancer Research Center, Seattle, WA USA; 10 Nuffield Department of Clinical Medicine, University of Oxford, Oxford, UK; 11 Department of Medicine, University of California, San Francisco, CA USA

**Keywords:** Vaccines, RNA vaccines

Correction to: *npj Vaccines* 10.1038/s41541-024-00818-y, published online 21 February 2024

In the original version of this Meeting Report, the incorrect statement “Out of nine HIV-1 vaccine efficacy trials completed so far, all but one demonstrated correlates of protection^4^” was inserted by mistake. The correct statement should have been “Out of nine HIV-1 vaccine efficacy trials completed so far, only one demonstrated correlates of protection^4^”. The correct statement is now shown in both the PDF and HTML versions of this Meeting Report.

